# H3K36 methyltransferase NSD1 is essential for normal B1 and B2 cell development and germinal center formation

**DOI:** 10.3389/fimmu.2022.959021

**Published:** 2022-11-30

**Authors:** Sulan Zhai, Min Cao, Han Zhou, Huamin Zhu, Tongchang Xu, Yuliang Wang, Xiaoming Wang, Zhenming Cai

**Affiliations:** ^1^ Department of Immunology, Key Laboratory of Immune Microenvironment and Diseases, Nanjing Medical University, Nanjing, China; ^2^ Reproductive Medicine Centre, Changzhou No. 2 People’s Hospital, The Affiliated Hospital of Nanjing Medical University, Changzhou, China; ^3^ State Key Laboratory of Reproductive Medicine, Nanjing Medical University, Nanjing, China; ^4^ National Health Commission (NHC) Key Laboratory of Antibody Technique, Nanjing Medical University, Nanjing, China

**Keywords:** NSD1, B2 cells, germinal center, antibody, B1 cells

## Abstract

B cells, which consist of two well-defined populations: B1 and B2 cells, which can produce antibodies that are essential for host protection against infections, through virus neutralization, opsonization and antibody-dependent cellular cytotoxicity. Epigenetic modifications, such as DNA methylation and histone modification could regulate immune cell differentiation and functions. In this study, we found a significant reduction of GC response in the B cell specific knockout of H3K36 methyltransferase NSD1 (Mb1-Cre^+^ NSD1^fl/fl^, NSD1^B KO^) mice compared with the wildtype control (Mb1-Cre^+^ NSD1^+/+^, NSD1^B WT^). We also demonstrated reduced production of high-affinity antibody, but increased production of low-affinity antibody in the NSD1^B KO^ mice. Further analysis revealed that loss of NSD1 promoted the development of B1 cells by increasing the expression of Rap1b and Arid3a. In conclusion, our data suggest that NSD1 plays an important role in regulation the development of B1 and B2 cells, and the process of germinal center formation and high-affinity antibody production.

## Introduction

B cells could produce antibodies which are essential for host protection against infections, through opsonization of pathogens for efficient phagocytosis by macrophages, virus neutralization and antibody-dependent cellular cytotoxicity (1, 2). The mature naive B cell repertoire consists of two well-defined populations: B1 and B2 cells. B1 cells, which were discovered in 1983 (3), mainly originated from the fetal liver and primarily located in the peritoneal cavity (4). They were an innate-like B lymphocyte subset and one of the main producers of natural antibodies, mainly IgM and IgG3, which could provide the first line of defence against a number of virus and bacteria (5–7). B1 cells were further subdivided into B1a and B1b cells. B1a cells derived from B1 progenitors/precursors in the fetal and neonatal livers and were maintained by self-renewal throughout adulthood, whereas B1b cells were differentiated both from the fetal liver and adult bone marrow (BM) B lymphopoiesis (8, 9). B2 cells were differentiated from the bone marrow and could be further divided into follicular B (FOB) and marginal zone B (MZB) cells (10, 11). The germinal center (GC) response, which was mainly mediated by follicular B cells, was the key requirement for mounting a long-term humoral immunity. GCs, which were formed in the center of the B cell follicles of secondary lymphoid organs, produce a group of mutated B cells which were then selected, based on the affinity, to proliferate and differentiate into plasma cells that could secret high-affinity antibody and memory B cells (12, 13). Abnormal development and distribution of B cells could result in primary immune deficiency, autoimmune diseases and even B cell malignancies (14).

*NSD1* (nuclear receptor SET (su(var)3–9, enhancer-of-zeste, trithorax) domain containing protein-1), which is isolated and characterized in 2001, belongs to the NSD protein lysine methyltransferase (KMT) family (15). This family has three members, NSD1 (KMT3B), NSD2 (WHSC1/MMSET) and NSD3 (WHSC1L1), which could regulate the expression of target genes through methylation of lysine 36 on histone H3 (H3K36) (16). NSD1 could bind various promoter elements to regulate transcription *via* interactions with H3K36 methylation and RNA polymerase II (17). Loss of function, heterozygous, or truncating mutations of NSD1 have been reported to be associated with two autosomal dominant genetic diseases, the Sotos syndrome (18, 19) and Beckwith–Wiedemann syndrome (20). NSD1 is associated with the development of acute myeloid leukemia (AML) (21), head and neck squamous cell carcinoma (22), neuroblastoma and glioma (23). NSD1 could also regulate the activity of NF-κΒthrough direct methylation of RELA, a component of NF-κΒ at lysines K218 and K221 (24), which suggests NSD1 may be a mediator of the inflammatory responses.

In previous study, we identified H3K36me2 methyltransferase Nsd2 was required for GC B cell adhesion to follicular dendritic cell expressed adhesion molecules (25). However, the role of NSD1 in immune response, especially in the development of B cells and the process of GC formation is unclear. In this work, we used B cell specific NSD1 knockout (NSD1^B KO^) mice to reveal the function of NSD1 in the development of B cells and GC formation. We found out that NSD1 did not affect the development of B2 cells in bone marrow, but was required for the generation of follicular B cells in the spleen. The loss of NSD1 in B cells reduced the formation of GC and production of high-affinity antibody, but increased the production of low-affinity antibody. Further analysis revealed that the loss of NSD1 promoted the development of B1 cells in the peritoneal cavity and spleen by increasing the expression of Rap1b and Arid3a.

## Materials and methods

### Generation of NSD1 conditional knockout mice

To generate NSD1 conditional knockout mice, a loxp site and aFNFL (Frt-Neo-Frt-Loxp) cassette were engineered to flank exon 6-8 of the NSD1 gene to generate the “floxed” NSD1 allele (**Supplementary Figure 1A**). Genomic DNA extracted from mice tails were used for genotyping by polymerase chain reaction (PCR) (**Supplementary Figure 1B**). The Mb1-Cre (CD79a-Cre) mice were purchased from The Jackson Laboratory.

All the mice were housed in a specific pathogen–free environment and animal protocols were reviewed and approved by the Institutional Animal Care and Use Committee (IACUC) of Nanjing Medical University. All the mice were maintained on C57BL/6J background and the control mice were littermate control. 7 – 9 weeks old male and female mice were used for experiments, except using 1 week old mice to explore the early development of B1 cells in the spleen.

### Flow cytometry

Bone marrow, spleen, mesenteric lymph node (mLN) and Peyer’s Patch (PP) cells were isolated as described previously (26). In brief, cells were stained with anti-B220 (BioLegend), anti-CD11b (BD Biosciences), anti-CD43 (eBioscience), anti-CD24 (BioLegend), anti-BP1 (eBioscience), anti-IgD (BioLegend), anti-IgM (BioLegend), anti-CD93 (eBioscience) and anti-CD23 (BioLegend) for B cell development staining. For B1 and B2 cell development staining, cells were stained with anti-B220 (BioLegend), anti-CD19 (BioLegend), anti-CD43 (eBioscience), anti-CD23 (BioLegend) and anti-CD5 (BioLegend). For FOB and MZB cell staining, cells were stained with anti-B220 (BioLegend), anti-CD19 (BioLegend), anti-CD21 (BD Biosciences) and anti-CD23 (BioLegend).Cells were stained with anti-B220 (BioLegend), anti-Gl7 (BioLegend), anti-CD95 (Fas) (BD Biosciences), anti-CD86 (BioLegend), and anti-CXCR4(BioLegend) for GC B cell staining and cells were stained with anti-B220 (BioLegend), anti-MHC II (eBioscience) and anti-CD86 (BioLegend) for B cell activation staining.

### Immunization

For induction of germinal centers, mice were immunized i.p. with 2×10^8^ sheep red blood cells (SRBCs). For serum immunoglobulin assay, mice were immunized i.p with 100 mg of NP-KLH or NP-Ficoll which were precipitated with aluminum hydroxide gel adjuvant (Accurate Chemical and Scientific).

### ELISA

An ELISA assay was performed as described previously (27). In brief, we coated the plates with NP2-BSA (2.5 mg/ml) or NP30-BSA (2.5 mg/ml) at 4°C overnight, washed the plates with PBST for three times, and blocked them with 2% BSA in PBST for 2 h at room temperature. Then, the serum from the mice which were immunized with NP-KLH on day 7, 14 and 21, or NP-Ficoll on day 7 were diluted with 1% BSA in PBS, added into the plates, and incubated at room temperature for 2 h. Then the plates were washed with PBST for 6 times and incubated with goat anti-mouse IgG1-HRP, goat anti-mouse IgG3-HRP, or goat anti-mouse IgM-HRP (Southern Biotech) for 1 h at room temperature. Finally, the plates were washed with PBST for 6 times, developed with TMB (Vector Laboratories), stopped, and read at 450 and 570 nm using a BioTek plate reader.

### RNA-seq

B220^+^ B cells were sorted from the spleen of NSD1^B WT^ and NSD1^B KO^ mice with a BD Aria II. Sorting efficiency was tested, and only cells with high purity (>98%) were used for RNA extraction and library construction. Sequencing were done by BGI Co., Ltd. on an Illumina Hiseq platform and 150bp paired-end module. Then we mapped the sequence reads to mm10 reference genome using Bowtie2 software and Fragments Per Kilobase per Million were calculated with Cufflinks. A threshold of fold change > 1.2 and p < 0.05 was used to determine the differential expression of genes.

### Cell sorting and mRNA analysis

B220^+^ B cells or B220^+^ Gl7^+^ Fas^+^ GC B cells from the spleen of NSD1^B WT^ and NSD1^B KO^ mice were sorted into PBS containing 0.5% FBS before RNA extraction with a BD Aria II. cDNA was synthesized with HiScript III 1st Strand cDNA Synthesis Kit (Vazyme Biotech Co., Ltd.) followed by PCR with primers 5’- CTCAGCAGCAGATCCCAAT -3’ and 5’- CTTCCTGAGGCGTTTCTTCT -3’ for *NSD1* (181-bp PCR product), 5’-AAATGCGACTTGGAAGATGA -3’ and 5’-TTCCCAGGCACTGGAGTT -3’ for *Rap1b* (173-bp PCR product), 5’-TGGACCTTTGAGGAGCAGTT-3’ and 5’-GATGGAGGTAGGCAGGTTGA-3’ for *Arid3a* (255-bp PCR product), and 5’-TCTGCTACGTGGTGAAGAGGAG-3’ and 5’-CCAGTCTGAGATGTAGCGTAGG-3’ for *AICDA* (119-bp PCR product). The *HPRT* gene was used as a control to generate a 312-bp PCR product with primers 5’-GGGGGCTATAAGTTCTTTGC-3’ and 5’-TCCAACACTTCGAGAGGTCC-3’.

### Immunofluorescence staining

For immunofluorescence staining, cryosections of the spleens from SRBCs immunized NSD1^B WT^ and NSD1^B KO^ mice were fixed with ice-cold acetone for 10 mins at room temperature. Then the sections were incubated with 2% BSA for 2 hr at room temperature. After that, sections were incubated with Gl7-FITC (BioLegend) and IgD-PE (BioLegend) for 2 hr at room temperature then incubated with DAPI for 5 mins at room temperature. Images were captured using OLYMPUS BX53 microscope.

### Mutation analysis

The mutation analysis was performed as described (25, 27). In brief, on day 21 after NP-KLH immunization, 30,000 GC B cells from NSD1^B WT^ and NSD1^B KO^ mice were sorted and pooled together, respectively, for genomic DNA extraction using a DNA Microprep kit (QIAGEN Cat: #56304). After nested PCR amplification, the VH186.2 H chains and JH4 were sequenced and analyzed with Vector NTI software to determine the frequency of somatic mutations. VH186.2 sequences and JH4 intronic sequences were analyzed with ImMunoGeneTics (IMGT) V-QUEST (http://www.imgt.org/) and only sequences of good quality were considered for mutation analysis.

### Statistical analysis

Student’s t tests were used for statistical analysis. The data in figures are displayed as the mean ± SEM unless otherwise indicated. *P*-values are denoted in figures by *, *P* < 0.05; **, *P* < 0.01 and ***, *P* < 0.001.

## Results

### The effects of NSD1 deficiency on B cell development

Mice carrying floxed NSD1 alleles (NSD1^fl/fl^) were mated with Mb1-Cre transgenic mice to specifically remove NSD1 in B cells. Q-PCR analysis confirmed the significantly decreased expression of NSD1 in NSD1 B cell conditional knockout (Mb1-Cre^+^ NSD1^fl/fl^, NSD1^B KO^) mice (**Supplementary Figure 1C**). Western blot analysis also confirmed that the level of H3K36me2 decreased significantly in NSD1^B KO^ mice compared with the WT (Mb1-Cre^+^ NSD1^+/+^, NSD1^B WT^) mice (**Supplementary Figure 1D**).

**Figure 1:**
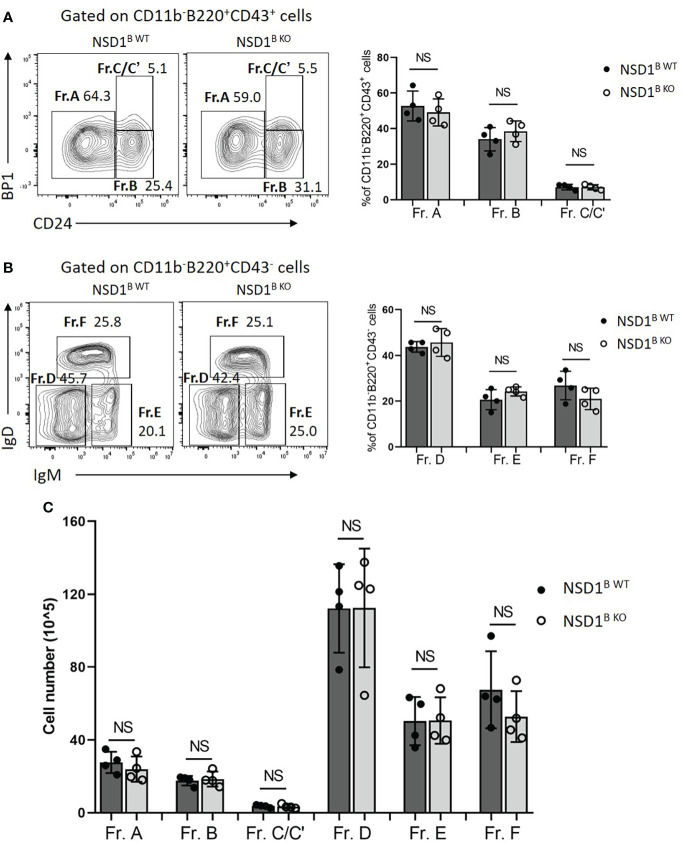
Loss of NSD1 did not affect the development of B cells in bone marrow. The development B cell were analysed in the bone marrow from WT (NSD1^B WT^) and NSD1 B cell deficient (NSD1^B KO^) mice. **(A)**. Hardy fractions (Fr. A CD24^-^Bp1^-^; Fr. B CD24^+^Bp1^-^ and Fr. C/C’ CD24^+^Bp1^+^) were analyzed in the CD11b^-^B220^+^CD43^+^ cells. **(B)**. Hardy fractions (Fr. D IgD^-^IgM^-^; Fr. E IgD^+^IgM^-^ and Fr. F IgD^+^IgM^+^) were analyzed in the CD11b^-^B220^+^CD43^-^ cells. **(C)**. The cell number of Fr. A - F in the bone marrow from the NSD1 ^B WT^ and NSD1 ^B KO^ mice. (n = 4). NS, no significant differences.

To identify whether NSD1 deficiency affects B cell development, we first detected the development of pro-, pre-, immature, and mature B cells in the bone marrow from NSD1^B WT^ and NSD1^B KO^ mice by flow cytometry based on specific B cell surface markers (28). And we found out that loss of NSD1 did not influence the development of the CD43^+^ pro-B cells (Fr. A CD24^-^Bp1^-^; Fr. B CD24^+^Bp1^-^ and Fr. C/C’ CD24^+^Bp1^+^), andthe CD43^-^ pre-B (Fr. D IgD^-^IgM^-^), immature B (Fr. E IgD^+^IgM^-^), and mature B cells (Fr. F IgD^+^IgM^+^) (**Figures 1A-C**).

**Figure 2:**
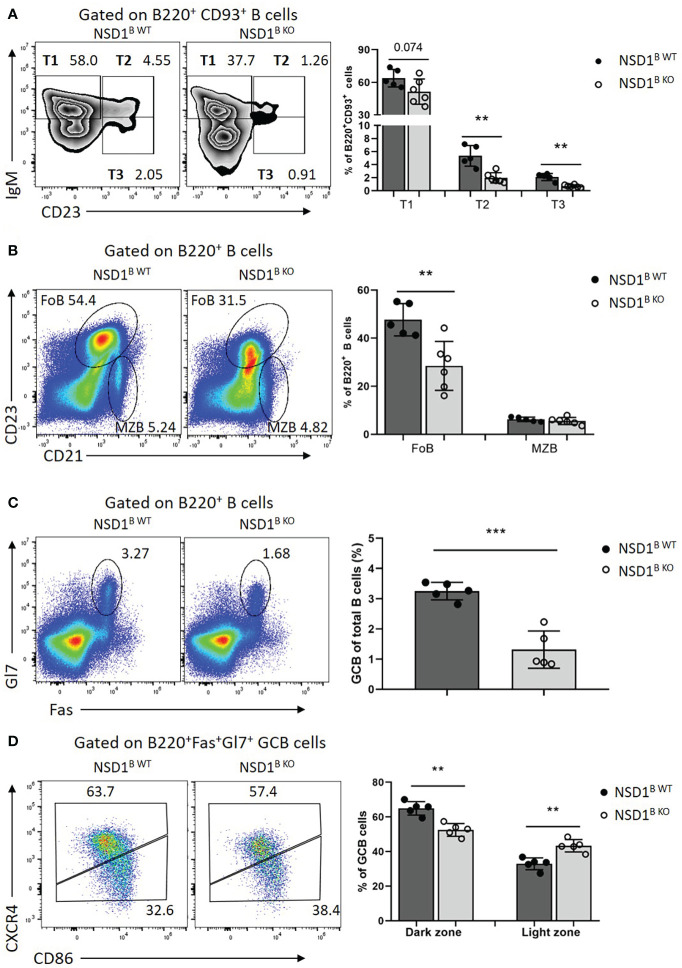
NSD1 is involved in the development of B cells and GC formation in spleen **(A)**. T1 (B220^+^CD93^+^IgM^high^CD23^-^), T2 (B220^+^CD93^+^IgM^high^CD23^+^) and T3 (B220^+^CD93^+^IgM^low^ CD23^+^) in the spleen from NSD1^B WT^ and NSD1^B KO^ mice. **(B)**. The distribution of follicular B (FOB, B220^+^CD23^+^CD21^low^) cell and marginal zone B (MZB, B220^+^CD23^-^CD21^high^) cell in the spleen from NSD1^B WT^ and NSD1^B KO^ mice. **(C)**. Germinal center B (B220^+^Gl7^+^Fas^+^) cells in the spleen from NSD1^B WT^ and NSD1^B KO^ mice 7 days after SRBCs immunization. **(D)**. The dark zone (DZ, CXCR4^+^CD86^-^) and light zone (LZ, CXCR4^-^CD86^+^) GC B cells in the spleen from NSD1^B WT^ and NSD1^B KO^ mice 7days after SRBCs immunization. **P < 0.01; ***P < 0.001. (n = 5 or 6).

Then the effect of NSD1 deletion on B cell compartments in the spleen, in which immature B cells undergo transitional stages to become follicular B (FOB) cells or marginal zone B (MZB) cells were inspected (10). And we found significant decrease of T2 (B220^+^CD93^+^IgM^hi^CD23^+^) and T3 (B220^+^CD93^+^ IgM^low^ CD23^+^) cells, and a slight decrease of T1 (B220^+^CD93^+^IgM^hi^CD23^-^) cells in the spleen from NSD1^B KO^ mice (**Figure 2A**). There was also a significant decrease of follicular cells (B220^+^CD23^+^CD21^low^) in the spleen of NSD1^B KO^ mice, but not marginal zone B cells (B220^+^CD23^-^CD21^high^) (**Figure 2B**). Taken together, our results demonstrated that NSD1 was required for the development of follicular B cells in the spleen, but was unessential for early B cell development in the bone marrow.

### NSD1 is involved in GC formation and antibody production

Then the NSD1^B WT^ and NSD1^B KO^ mice were immunized with SRBCs and the germinal center B (GC B, B220^+^Gl7^+^Fas^+^) cells in the spleen from these mice were analyzed 7 days after immunization. The frequency of GC B cells decreased significantly in the NSD1^B KO^ mice compared with the WT control (**Figure 2C**). We also found decreased dark zone (DZ, CXCR4^+^ CD86^-^), but increased light zone (LZ, CXCR4^-^CD86^+^) of GC B cells in the spleen from NSD1^B KO^ mice in further analysis (**Figure 2D**). The decreased GC formation in the spleen was also confirmed by immunofluorescent staining (**Supplementary Figure 2A**). And the germinal center defect was caused by impaired proliferation in the NSD1^B KO^ mice (**Supplementary Figure 2B**). Decreased GC formation in the mesenteric lymph node (mLN) and Peyer’s Patch (PP) was also found in the NSD1^B KO^ mice (**Supplementary Figures 2C, D**). We also analysed the expression of AICDA in the germinal center B cells sorted from the spleen of NSD1^B WT^ and NSD1^B KO^ mice 7 days after SRBCs immunization by Q-PCR and found out that the expression of AICDA decreased significantly in the NSD1^B KO^ mice (**Supplementary Figure 3A**). On day 21 after immunization, GC B cells from NSD1^B WT^ and NSD1^B KO^ mice were sorted and pooled together, respectively, for genomic DNA extraction. After nested PCR amplification, the VH186.2 H chains and JH4 were sequenced to determine the frequency of somatic mutations. And we found decreased frequency with high mutation number of JH4 (≥1) and VH186 (≥10) in the GC B cells sorted from the spleen of NSD1^B KO^ mice. The frequency of GC B cells with higher-affinity mutations (W33L/K59R/99G) (29) also decreased in the NSD1^B KO^ mice, but not significantly (**Supplementary Figures 3B, C**). These results suggested that NSD1 was essential for the GC formation.

**Figure 3:**
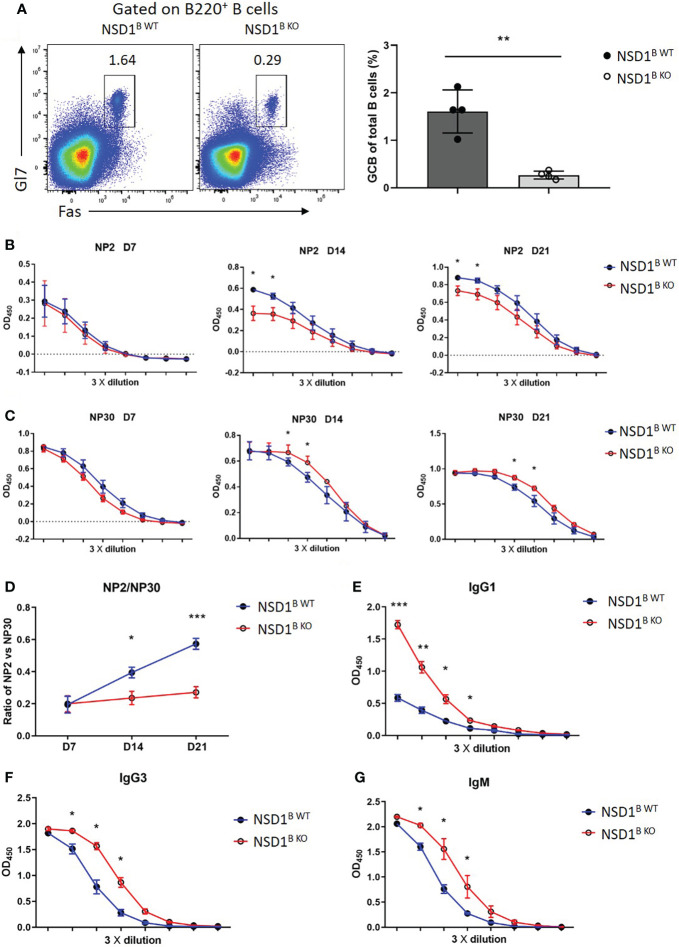
Loss of NSD1 affected the production of high- or low-affinity antibodies **(A)**. Germinal center B (GC B, B220^+^Gl7^+^Fas^+^) cells in the spleen from NSD1^B WT^ and NSD1^B KO^ mice 21 days after NP-KLH immunization. (n = 4). **(B, C)**. NP2-specific **(B)** and NP30-specific **(C)** IgG antibody in serum were measured at day 7, 14 and 21 after NP-KLH immunization. Quantification data are shown as mean ± SD. **(D)**. Ratios of NP2/NP30 were calculated with raw optical density (OD) value in linear range. Statistical analysis was done with two-way ANOVA. **(E)**. T cell independent NP30-specific IgG1 antibody in serum was measured at day 7 after NP-Ficoll immunization. Quantification data are shown as mean ± SD. **(F, G)**. T cell independent IgG3 and IgM antibody in serum were measured at day 7 after NP-Ficoll immunization. Quantification data are shown as mean ± SD. *P < 0.05, **P < 0.01 and ***P < 0.001.

Germinal centers function as the site of B cell clonal expansion, somatic hypermutation, and affinity-based selection, which results in the generation of high-affinity antibodies (30). Then we monitored the humoral immune response to the thymus-dependent (TD) antigen NP-KLH in alum by measuring the levels of NP2-specific or NP30-specific antibodies in the serum at different days post-immunization by ELISA. The frequency of GC B cells decreased significantly in the NSD1^B KO^ mice 21 days after immunization, which was consistent with SRBCs immunization (**Figure 3A**). The high-affinity NP2-specific antibody decreased significantly at day 14 and 21 in the serum from NSD1^B KO^ mice, but low-affinity NP30-specific antibody increased (**Figures 3B, C**). Ratios of NP2/NP30 also decreased in the serum from NSD1^B KO^ mice (**Figure 3D**). We then immunized the mice with the thymus-independent (TI) antigen NP-Ficoll and found out that the levels of IgG1, IgG3 and IgM antibodies increased significantly in the serum from NSD1^B KO^ mice than the NSD1^B WT^ mice (**Figures 3E-G**). These results illustrated that the loss of NSD1 in B cell reduced the formation of GC and production of high-affinity antibody, but increased the production of low-affinity antibody.

### Loss of NSD1 promoted the development of B1 cells

**Figure 4:**
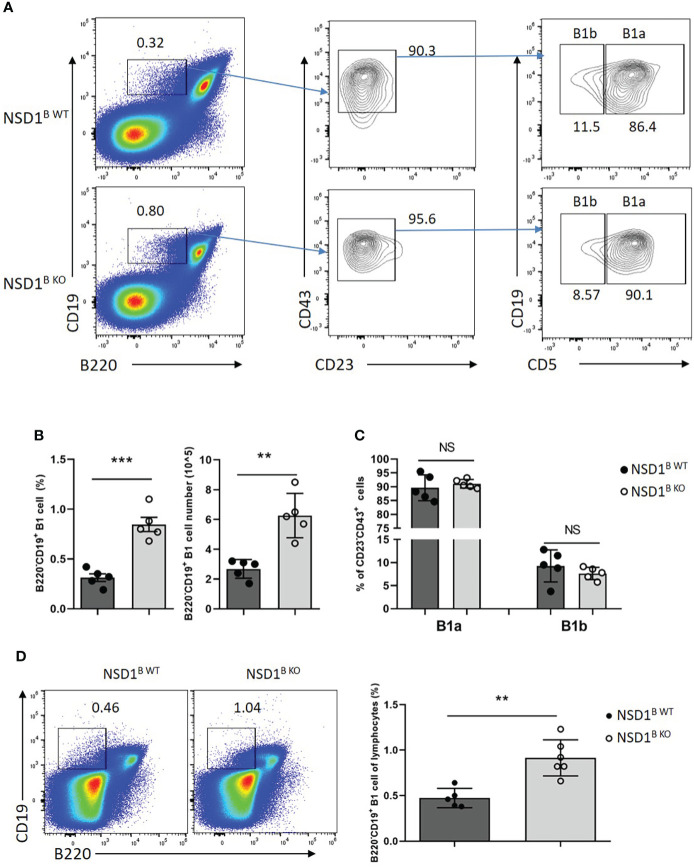
NSD1 deficiency promoted the development of B1 cells in spleen **(A-C)**. B1 cells (B220^+^CD19^-^), B1a (B220^+^CD19^-^CD43^+^CD23^-^CD5^+^) and B1b (B220^+^CD19^-^CD43^+^CD23^-^CD5^-^) in the spleen from NSD1^B WT^ and NSD1^B KO^ mice. (n = 5). **(D)**. B1 cells (B220^+^CD19^-^) in the spleen from one week old NSD1^B WT^ and NSD1^B KO^ mice. (n = 5 or 6). **P < 0.01, ***P < 0.001. NS, no significant differences.

Then we want to determine the source of increased low-affinity antibody in NSD1^B KO^ mice. It was reported that plasma cells derived from B1 cells are the main source of antibodies of the IgM (3). We analyzed the distribution of B1 cells in the spleen and found increased B1 cells (B220^+^CD19^-^) from NSD1^B KO^ mice than the NSD1^B WT^ mice (**Figures 4A, B**). But there was no difference of the distribution of B1a (B220^+^CD19^-^CD5^+^) and B1b (B220^+^CD19^-^CD5^-^) cells (**Figure 4C**). In addition, the increase of B1 cells in the spleen was present in NSD1^B KO^ mice when they were only one week old (**Figure 4D**).

**Figure 5:**
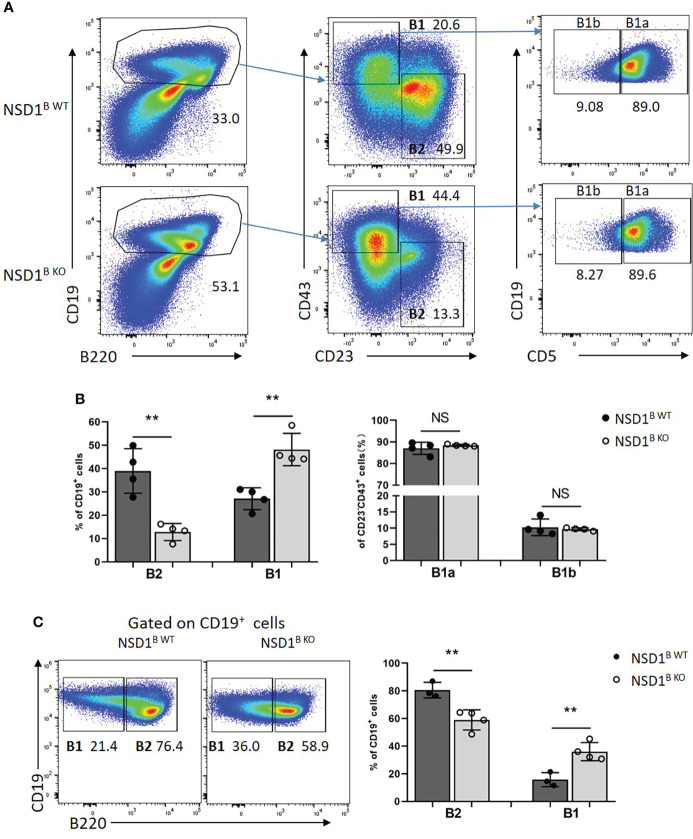
Loss of NSD1 increased the development of B1 cells in peritoneal cavity **(A, B)**. The distribution of B1 (CD19^+^CD43^+^CD23^-^), B2 (CD19^+^ CD43^-^CD23^+^) cells, B1a (CD19^+^CD43^+^CD23^-^CD5^+^) and B1b (CD19^+^CD43^+^CD23^-^CD5^-^) in the peritoneal cavity from NSD1^B WT^ and NSD1^B KO^ mice. **(B)**. The distribution of B1 (CD19^+^B220^-^) and B2 (CD19^+^B220^+^) cells in the peritoneal cavity from NSD1^B WT^ and NSD1^B KO^ mice. (n = 3 or 4). **P < 0.01. NS, no significant differences.

B1 cells are primarily located in the peritoneal cavity (31). We analyzed the distribution of B1 (CD19^+^B220^-^) and B2 (CD19^+^B220^+^) cells in the peritoneal cavity from NSD1^B WT^ and NSD1^B KO^ mice by flow cytometry, and found increased B1 but decreased B2 cells in NSD1^B KO^ mice, but there was no difference of the distribution of B1a and B1b cells (**Figures 5A, B**). The different distribution of B1 and B2 cells in the peritoneal cavity between NSD1^B WT^ and NSD1^B KO^ mice was also confirmed by CD19 and B220 staining (**Figure 5C**). These results revealed that the loss of NSD1 promoted the development of B1 cells in the peritoneal cavity and spleen.

### Rap1b and Arid3a promoted the development of B1 cells in NSD1^B KO^ mice

**Figure 6:**
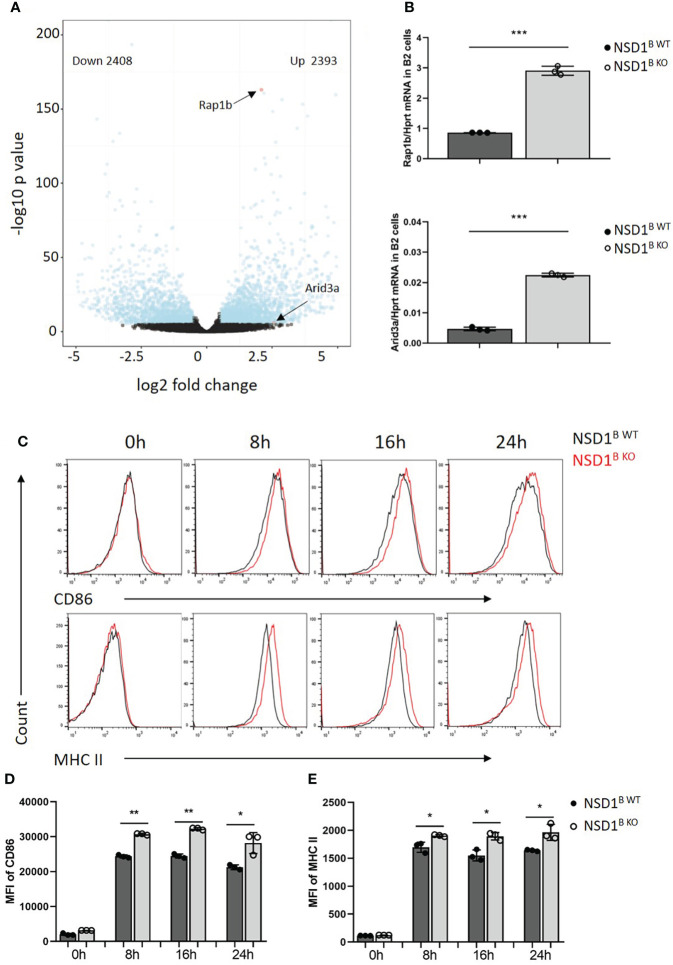
Rap1b and Arid3a promoted the development of B1 cells in NSD1 ^B KO^ mice **(A)**. RNA-seq analysis of B220^+^ B cells in the spleen from NSD1^B WT^ and NSD1^B KO^ mice. Differentially expressed genes (DEGs) (Padjust < 1e^-5^ and log2 [fold change] greater than 0.5 or less than -0.5) are shown in blue, and non-DEGs are shown in black. **(B)**. Q-PCR analysis of Rap1b (up) and Arid3a (down) expression in B220^+^ B cells. **(C-E)**. Histogram showing flow-cytometric analysis of CD86 and MHC II staining in B220^+^ B cells from spleen of NSD1^B WT^ and NSD1^B KO^ mice 8h, 16h and 24h after activation by IgM and CD40. Data are representative of 2 experiments. *P < 0.05, **P < 0.01, ***P < 0.001.

To explore the mechanism of increased B1 cells in NSD1^B KO^ mice, the B220^+^ B cells from the spleen of NSD1^B WT^ and NSD1^B KO^ mice were sorted and analyzed by RNA-seq. We found out 4801 differentially expressed genes (2408 up-regulated genes and 2393 down-regulated genes) between the NSD1^B WT^ and NSD1^B KO^ mice. Rap1b (32) and Arid3a (33, 34) have been reported to play key role in the progress of B1 cell development. And we found significantly increased expression of Rap1b and Arid3a in the B cells from NSD1^B KO^ mice than the NSD1^B WT^ mice (**Figure 6A**). The increased expression of Rap1b and Arid3a was also confirmed by Q-PCR (**Figure 6B**). And the pathways of the different expression genes were analysed by Gene Ontology (GO) enrichment and KEGG enrichment. Most DEGs were enriched in the biological processes of mRNA processing, ribonucleprotein complex biogenesis, ncRNA metabolic process, chromatin organization and RNA splicing; the cellular components of organelle inner membrane, mitochondrial inner membrane, nuclear speck, ribosome and mitochondrial matrix; and the molecular functions of transcription coregulator activity, ubiquitin-like protein transferase activity, DNA-binding transcription factor binding, catalytic activity acting on RNA, and ubiquitin-protein transferase activity. DEGs were mainly enriched in 15 KEGG pathways (multiple diseases, Amyotrophic lateral sclerosis, Alzheimer disease, Huntington disease, Parkinson disease, Prion disease and Salmonella infection) (**Supplementary Figure 4**).

On the other hand, it was reported that increasing strength of signals through B cell receptors promoted B-1 cell development in BCR transgenic mice (35, 36). Moreover, Kraus, M., et al. found out that Ig ^FF/FF^ mice, which responded less efficiently to T cell–dependent antigens, had a specific reduction of B1 in the peritoneal cavity (37). When the B cells from the spleen of NSD1^B WT^ and NSD1^B KO^ mice were treated with anti-IgM (1μg/ml) and anti-CD40 (5μg/ml), we found increased levels of activated marker CD86 and MHC II staining in B cells from NSD1^B KO^ mice than NSD1^B WT^ mice on 8h, 16h and 24h after activation (**Figures 6C–E**). These results indicated that B cells from NSD1^B KO^ mice had a stronger BCR signal than the control when they were activated by the same condition. Taken together, the increased expression of Rap1b and Arid3a, and a stronger BCR signal after activation promoted the development of B1 cells in NSD1^B KO^ mice.

## Discussion

In this study, we identified NSD1 plays an important role in regulation the development of B1 and B2 cells, and the process of germinal center formation and high-affinity antibody production.

B cells, which play crucial roles in both innate and adaptive immunity, are an important part of the immune system. The development of B cell occurs in an orderly fashion and is regulated by both intrinsic genetic programs and external cues such as cytokines which are present in the specific microenvironments of the fetal liver or bone marrow. Abnormities in each stage of the B cell development and maturation pathway could result in primary immunodeficiencies, autoimmune diseases and even B cell malignancies (14). The major stages of B cell development in the bone marrow include the hematopoietic stem cell (HSC), the multipotent progenitor (MPP), the common lymphoid progenitor (CLP), and then the progenitor B cell (pro-B cell), the precursor B cell (pre-B cell) and the immature B cell, which could be divided into fractions A, B, C, C′, D and E based on the Ig gene rearrangement status and the expression of different surface markers (38). In this study, we did not found difference of early B cell development in the bone marrow between the NSD1^B WT^ and NSD1^B KO^ mice, which indicated that NSD1 was not required for early B cell development in bone marrow.

Immature B cells have a short half-life and could be exported to the peripheral lymphoid organs, usually the spleen, where they complete the developmental program. Transitional B cells have a key role in linking bone marrow immature and peripheral mature B cells during the process of B2 cell maturation. Newly generated immature B cells are known as T1 B cells, which have the ability to recirculate throughout the body. After entering spleen follicles, T1 cells, which acquire cell surface expression of IgD, CD23 and CD21, and the ability to recirculate, but still carry markers of immaturity, are known as T2 B cells. T3, which is similar to T2 but a lower level of surface IgM, is a third non-proliferating transitional population (39). In this study, we found that the loss of NSD1 impaired the development of T2 and T3 B cells, follicular B cells in the spleen, but not the marginal zone B cells. We also revealed reduced GC formation in the spleen of NSD1^B KO^ mice after SRBCs immunization. When the mice were treated with TD antigen NP-KLH, NSD1^B KO^ mice shown decreased production of high-affinity antibody, but increased production of low-affinity antibody. These results revealed that NSD1 played an important role in the development and normal function of B2 cells.

B1 cells, which is associated with the production of low-affinity, poly-specific antibodies, comprise a unique subset of B cells involved in innate immunity, autoimmunity and immune regulation (40, 41). The increased B1 cells in the spleen and peritoneal cavity were found in NSD1^B KO^ mice, which may be the source of increased low-affinity antibody. When we immunized the mice with the thymus-independent antigen NP-Ficoll, the increased levels of low-affinity IgG1, IgG3 and IgM antibodies in the serum from NSD1^B KO^ mice were confirmed.

The strength of the BCR signal could affect the development of B1 cells. Mutations that disrupt BCR signal result in substantial depletion of the B1 subset while largely sparing B2 cells. On the other hand, mutations or transgenes that enhance BCR signal result in an expanded B1 compartment (6). In the current work, we found out that B cells from NSD1^B KO^ mice had a stronger BCR signal than the wild-type control when they were activated by anti-IgM and anti-CD40, which may promote the development of B1 cells.

Several genes have been identified as the master regulator of the genetic program that controls the development of B1 cells (33, 34, 42–44). Ishihara, S., et al. reported severe reduction of B1a cells in the peritoneal cavity, spleen and blood of Rap1a and Rap1b DKO mice (32). Retroviral transduction of adult bone marrow pro-B cells with Arid3a induced production of B1a B cells, and Arid3a shRNA could inhibit B1a generation from FL pro-B cells, revealing the key role of Arid3a in the switch from B2 to B1 development (34). We found significantly increased expression of Rap1b and Arid3a in the B cells from NSD1^B KO^ mice by RNA-seq and Q-PCR, which may be the cause of the increased B1 cells. However, the molecular mechanism by which NSD1 deletion in B cells leads to the increased expression of Rap1b and Arid3a remains unclear.

Collectively, this work indicated that NSD1 has an important role in the development of B1 and B2 cells, and the process of germinal center formation and antibody production. The increased expression of Rap1b and Arid3a induced by the loss of NSD1 and a stronger BCR signal after activation promoted the development of B1 cells, which make antibodies of low-affinity, in NSD1^B KO^ mice.

## Data availability statement

The datasets presented in this study can be found in online repositories. The names of the repository/repositories and accession number(s) can be found below: NCBI under accession ID: GSE208327.

## Ethics statement

The animal study was reviewed and approved by Institutional Animal Care and Use Committee (IACUC) of Nanjing Medical University. Written informed consent was obtained from the owners for the participation of their animals in this study.

## Author contributions

SZ, MC, XW and ZC conceptualized the project and designed the experiments. MC, SZ, HZ, HMZ, TX and ZC performed the experiments. YW, XW and ZC analyzed the data and wrote the manuscript. All authors contributed to the article and approved the submitted version.

## Funding

This work was supported by the National Natural Science Foundation of China (Grant No. 31970828 to XW and 81701542 to ZC), the National Key R&D Program of China (Grant No. 2018YFC1003900 to XW) and the Jiangsu Outstanding Young Investigator Program (Grant No. BK20200030 to XW).

## Conflict of interest

The authors declare that the research was conducted in the absence of any commercial or financial relationships that could be construed as a potential conflict of interest.

## Publisher’s note

All claims expressed in this article are solely those of the authors and do not necessarily represent those of their affiliated organizations, or those of the publisher, the editors and the reviewers. Any product that may be evaluated in this article, or claim that may be made by its manufacturer, is not guaranteed or endorsed by the publisher.

## Supplementary material

Supplementary Figure 1 The generation and identification of NSD1 conditional knockout mice **(A)**. Diagram showing the generation of “floxed” NSD1 allele (NSD1^fl/fl^) mice. **(B)**. Mouse genotyping of the altered NSD1 allele. **(C)**. The mRNA levels of NSD1 in sorted B220^+^ B cells from the spleen of NSD1^B WT^ and NSD1^B KO^ mice were analysed by Q-PCR. **(D)**. The levels of H3K36me2 and H3 in sorted B220^+^ B cells from the spleen of NSD1^B WT^ and NSD1^B KO^ mice were analysed by western blot.

Supplementary Figure 2 Loss of NSD1 affected GC formation in spleen, mesenteric lymph node and Peyer’s Patch A. Sections of spleens from NSD1^B WT^ and NSD1^B KO^ mice 7 days after SRBCs immunization were stained to detect follicular B cells (IgD) and GC B cells (Gl7). Bar, 500 μm. B. Cell cycle of GC B cells were analyzed by DNA content staining. (n=4) **(C, D)**. Germinal center B (B220^+^Gl7^+^Fas^+^) cells in the mesenteric lymph node **(C)** and Peyer’s Patch **(D)** from NSD1^B WT^ and NSD1^B KO^ mice 7 days after SRBC immunization. (n = 3). ** *P* < 0.01.

Supplementary Figure 3 Analysis of *AICDA* expression and SHM in NSD1^B WT^ and NSD1^B KO^ mice. **(A)**. The expression of *AICDA* in the germinal center B cells sorted from the spleen of NSD1^B WT^ and NSD1^B KO^ mice (n = 3). **(B)**. The frequency of JH4 and VH186.2 H chains mutations in the germinal center B cells sorted from the spleen of NSD1^B WT^ and NSD1^B KO^ mice. **(C)**. The frequency of GC B cells that acquired indicated higher-affinity mutations (W33L/K59R/99G) sorted from the spleen of NSD1^B WT^ and NSD1^B KO^ mice.

Supplementary Figure 4 Gene Ontology (GO) enrichment and KEGG enrichment analysis of the different expression genes. **(A)**. Gene Ontology enrichment analysis of the different expression genes. **(B)**. KEGG enrichment analysis of the different expression genes.

## Abbreviations

BM, bone marrow; FOB, follicular B cell; MZB, marginal zone B cell; GC, germinal center; mLN, mesenteric lymph node; PP, Peyer’s Patch; SRBCs, sheep red blood cells; BCR, B cell antigen receptor.
