# Supplementary figures and images for: H3K36 methyltransferase NSD1 is essential for normal B1 and B2 cell development and germinal center formation
Source: Front Immunol. 2022 Nov 30;13:959021. doi: 10.3389/fimmu.2022.959021 (PMC9750791; doi:10.3389/fimmu.2022.959021)

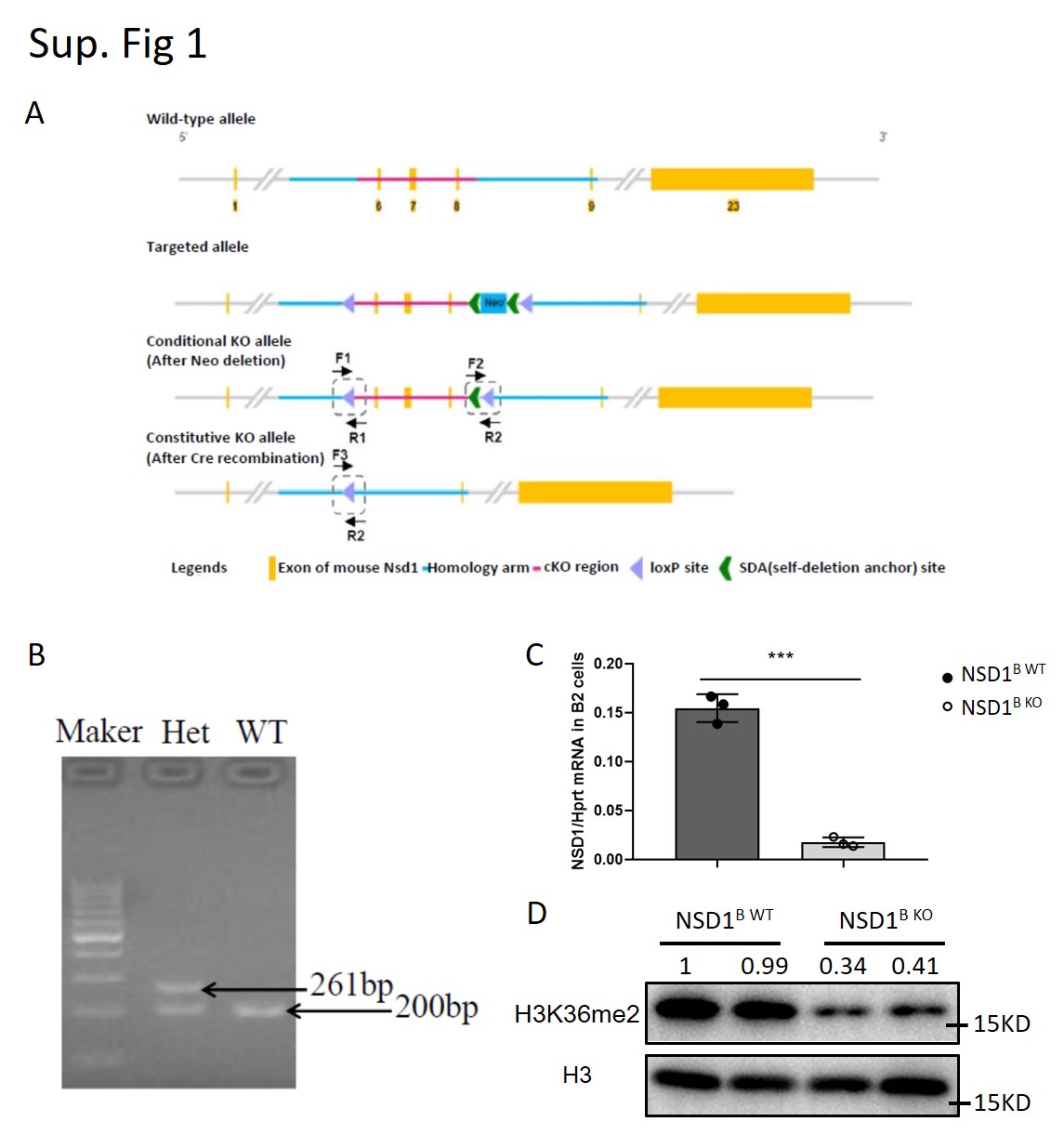

Supplement: Supplementary Figure 1 — The generation and identification of NSD1 conditional knockout mice (A). Diagram showing the generation of “floxed” NSD1 allele (NSD1fl/fl) mice. (B). Mouse genotyping of the altered NSD1 allele. (C). The mRNA levels of NSD1 in sorted B220+ B cells from the spleen of NSD1B WT and NSD1B KO mice were analysed by Q-PCR. (D). The levels of H3K36me2 and H3 in sorted B220+ B cells from the spleen of NSD1B WT and NSD1B KO mice were analysed by western blot. [file Image_1.jpeg]

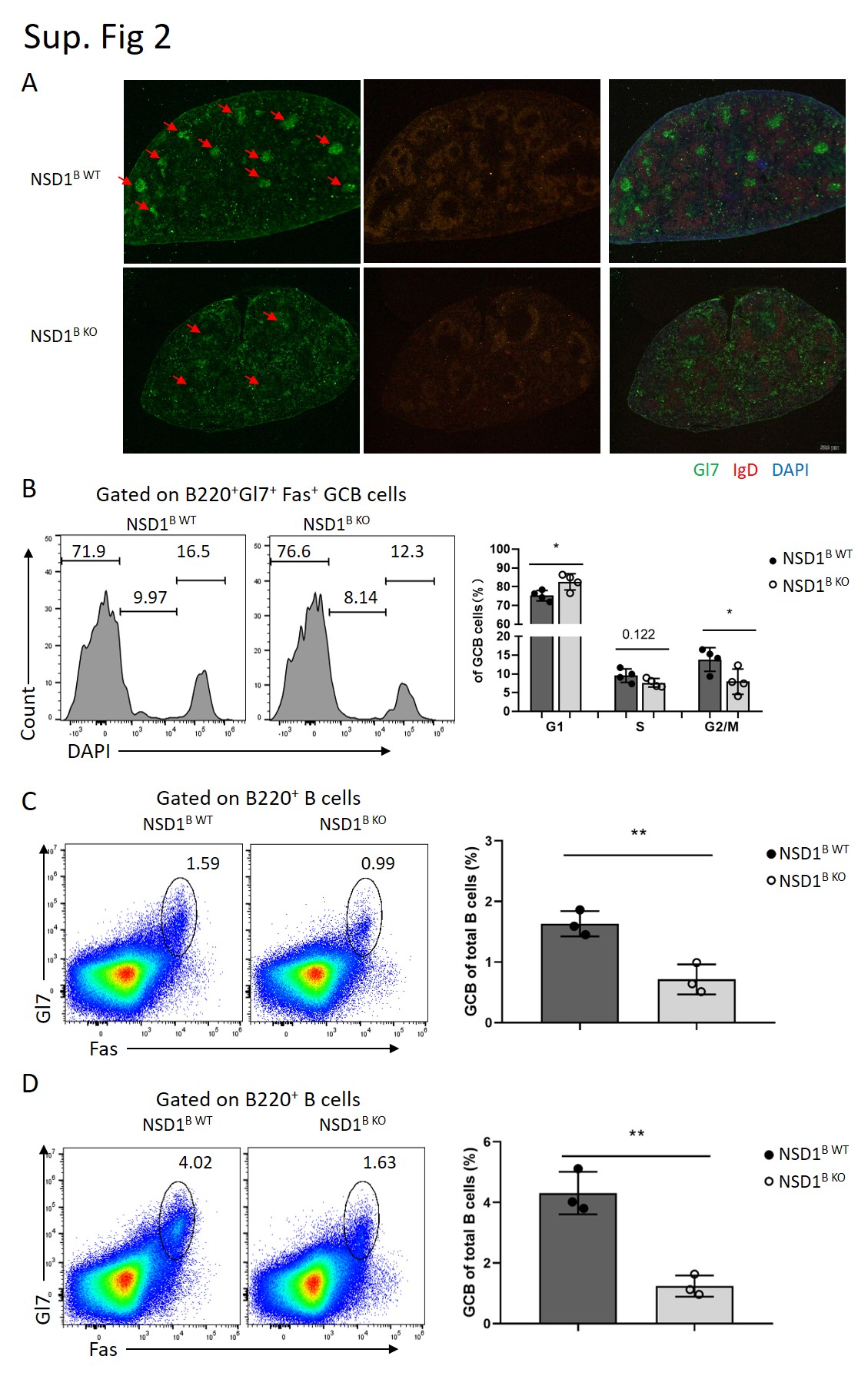

Supplement: Supplementary Figure 2 — Loss of NSD1 affected GC formation in spleen, mesenteric lymph node and Peyer’s Patch A. Sections of spleens from NSD1B WT and NSD1B KO mice 7 days after SRBCs immunization were stained to detect follicular B cells (IgD) and GC B cells (Gl7). Bar, 500 μm. B. Cell cycle of GC B cells were analyzed by DNA content staining. (n=4) (C, D). Germinal center B (B220+Gl7+Fas+) cells in the mesenteric lymph node (C) and Peyer’s Patch (D) from NSD1B WT and NSD1B KO mice 7 days after SRBC immunization. (n = 3). ** P < 0.01. [file Image_2.jpeg]

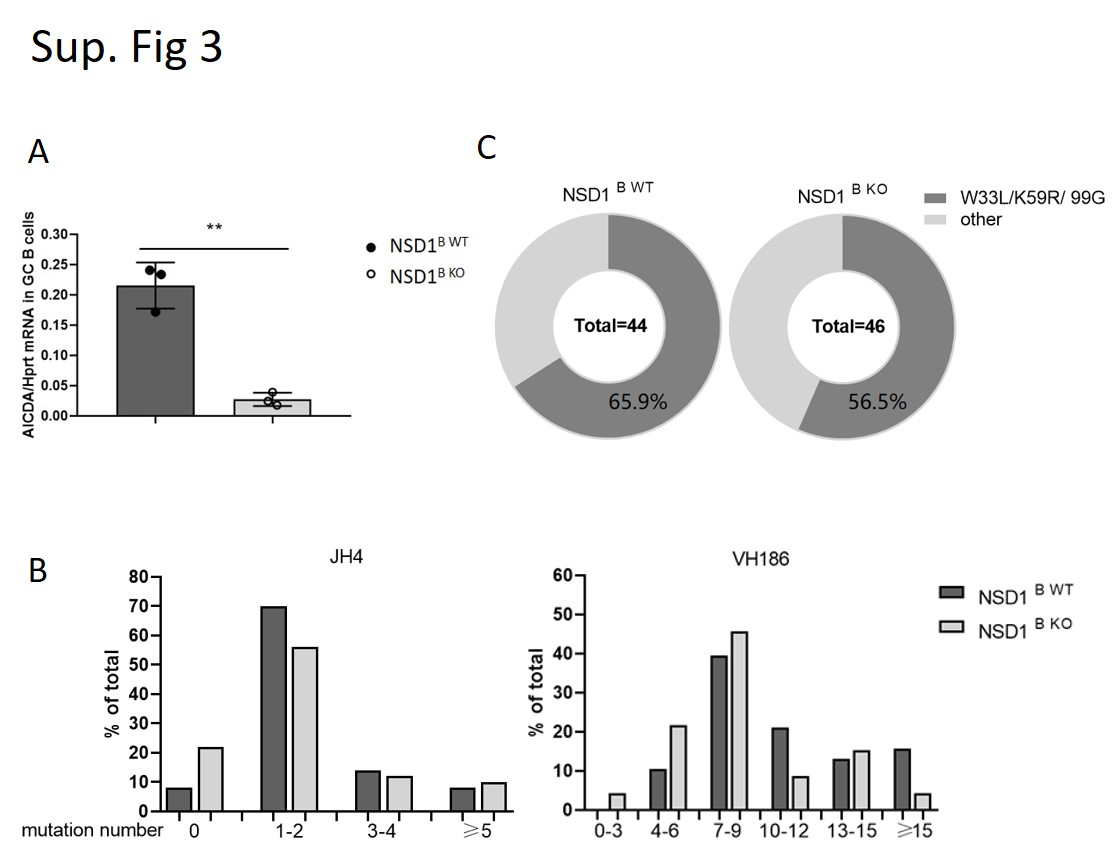

Supplement: Supplementary Figure 3 — Analysis of AICDA expression and SHM in NSD1B WT and NSD1B KO mice. (A). The expression of AICDA in the germinal center B cells sorted from the spleen of NSD1B WT and NSD1B KO mice (n = 3). (B). The frequency of JH4 and VH186.2 H chains mutations in the germinal center B cells sorted from the spleen of NSD1B WT and NSD1B KO mice. (C). The frequency of GC B cells that acquired indicated higher-affinity mutations (W33L/K59R/99G) sorted from the spleen of NSD1B WT and NSD1B KO mice. [file Image_3.jpeg]

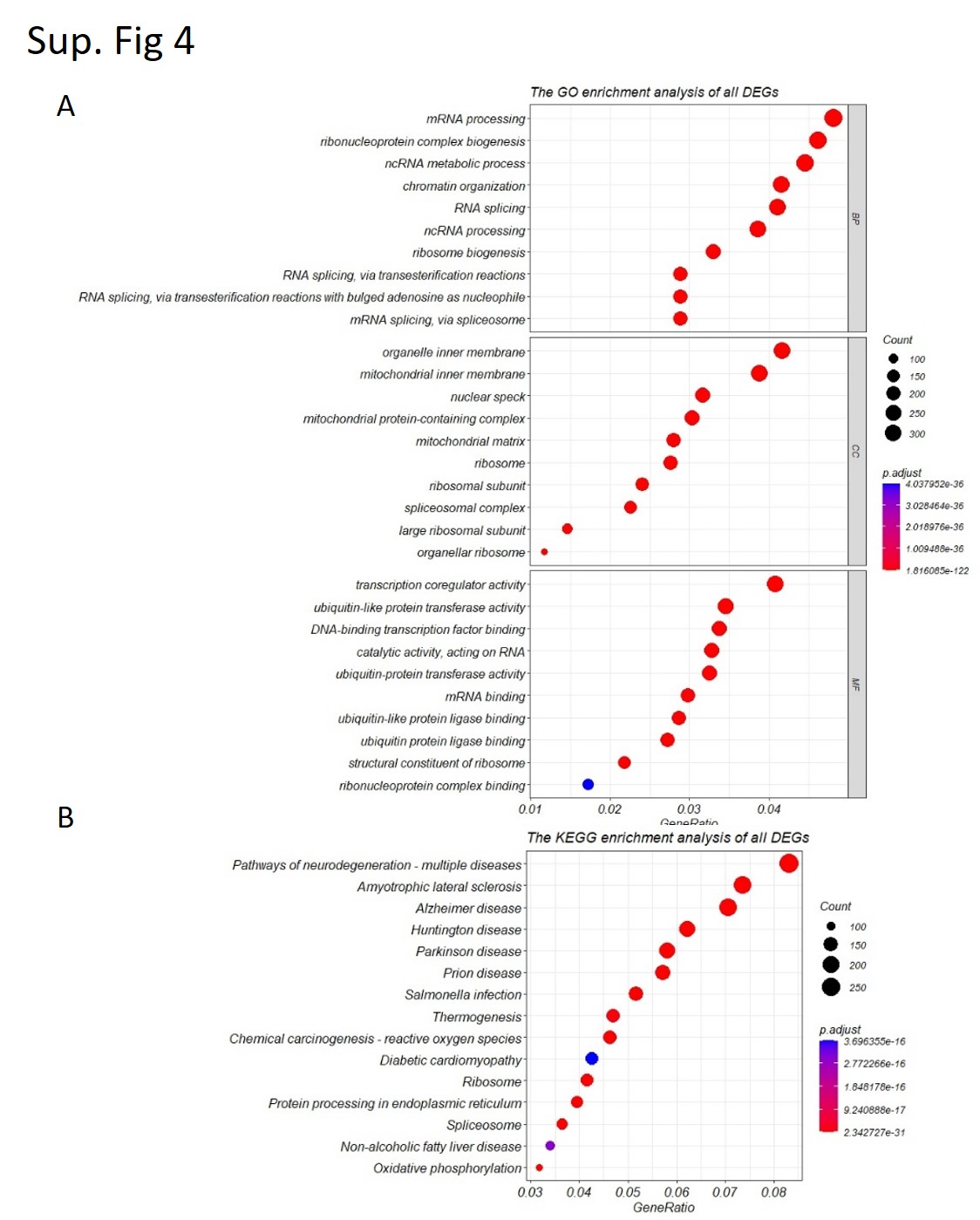

Supplement: Supplementary Figure 4 — Gene Ontology (GO) enrichment and KEGG enrichment analysis of the different expression genes. (A). Gene Ontology enrichment analysis of the different expression genes. (B). KEGG enrichment analysis of the different expression genes. [file Image_4.jpeg]
